# Life-threatening Bleed Secondary to Tumor Shrinkage Effectively Palliated with Radiotherapy

**DOI:** 10.7759/cureus.1386

**Published:** 2017-06-24

**Authors:** Michelle Tseng, Wanyi Yew, Anand Jeyasekharan, Balamurugan Vellayappan

**Affiliations:** 1 Radiation Oncology, National University Cancer Institute, National University Hospital Singapore; 2 Medical School, National University Hospital Singapore; 3 Medical Oncology, National University Cancer Institute, National University Hospital Singapore

**Keywords:** inverted sinonasal papilloma, radiotherapy, bleeding

## Abstract

Inverted papilloma is a typically benign, but locally aggressive tumor arising from the nasal cavity and paranasal sinuses. Malignant transformation can occur in up to 10% of cases. Although spontaneous tumor bleeding can occur with malignancies, hemorrhage secondary to tumor shrinkage has not been reported. We present a patient with metastatic squamous cell carcinoma (from inverted papilloma) who developed a life-threatening bleed shortly after chemotherapy initiation. She was managed successfully with life-saving palliative radiotherapy (RT), delivered based on clinical markup. She was subsequently re-treated with highly conformal RT and chemotherapy to achieve a marked clinical response without surgery.

## Introduction

Inverted papilloma of the nasal cavity is a benign epithelial neoplasm, which represents less than 4% of all nasal tumors [1]. Inverted papilloma has a locally destructive growth pattern with a tendency for local recurrence [2]. Malignant transformation can occur in up to 10% of cases, and distant metastases may involve the lung or brain [1]. In non-metastatic cases, surgery is the primary modality for management [1], with the consideration for adjuvant radiotherapy (RT) in certain cases. Systemic chemotherapy is utilized in the metastatic setting with a palliative intent. Doublet chemotherapy, with agents such as 5-flurouracil and cisplatin, achieve response rates of 20–30% [3]. Many of the head and neck squamous cell carcinoma overexpress epidermal growth factor receptor and show increased responses to regimens containing cetuximab [4]. Tumor bleed is commonly seen in locally progressive tumors, which have directly infiltrated blood vessels. However, tumor bleed secondary to rapid shrinkage has not been reported previously. We report on a patient who had a torrential bleed after initiation of multi-agent chemotherapy. This situation necessitated life-saving RT, which had to be delivered using an improvised set-up. 

## Case presentation

**Figure 1:**
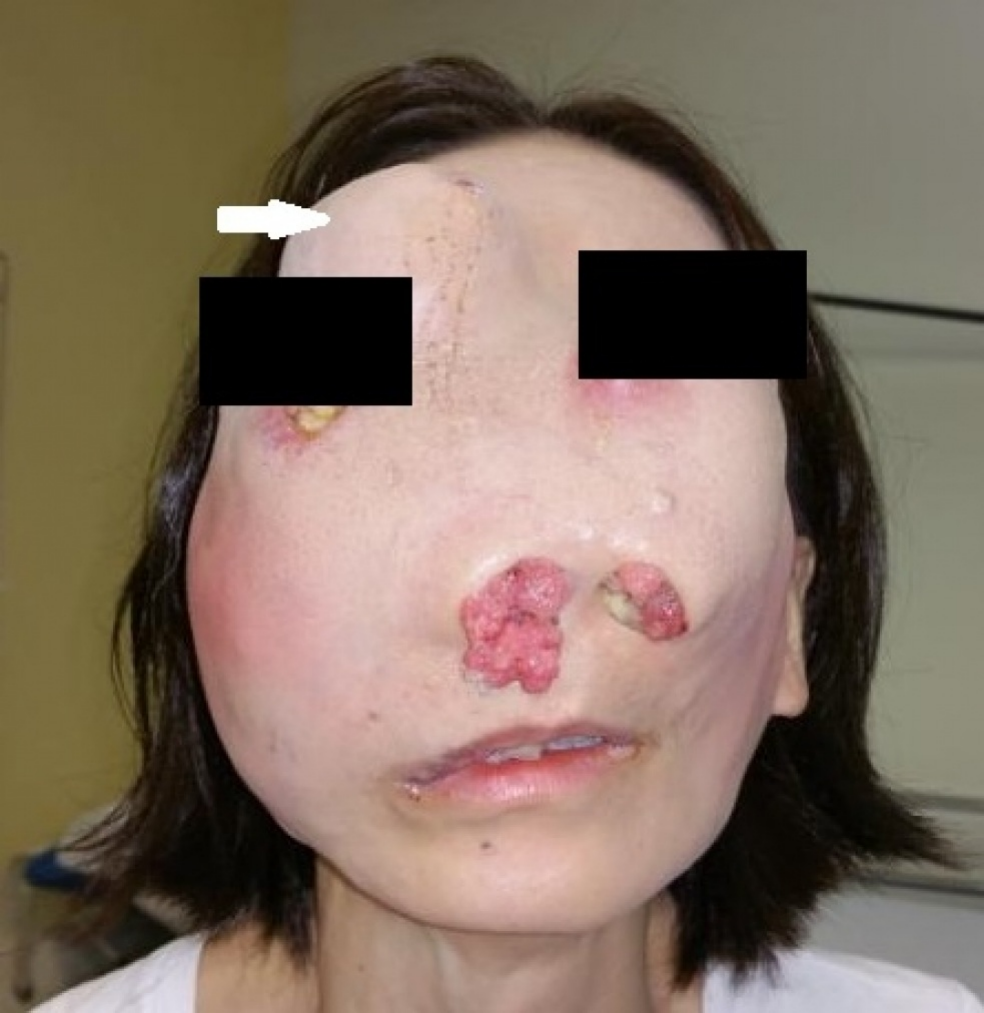
Pre-treatment clinical photograph Large exophytic tumor with protrusion through both nostrils. Tumor displaced both eyes laterally and caused ulceration of the overlying skin. Cystic component is noted above the right orbit (white arrow).

A 43-year-old Chinese female was first diagnosed in 2001 with an inverted papilloma involving the right nasal and orbital wall. She had declined surgical resection then. She re-presented in 2015 with disfiguring painful left facial swelling and headaches of a three-month duration. On examination, there was a large tumor causing distortion of her face with exophytic lesions protruding from her nostrils and involving the hard palate (Figure 1). Both eyes were displaced laterally, but visual acuity and visual fields were intact. 

**Figure 2:**
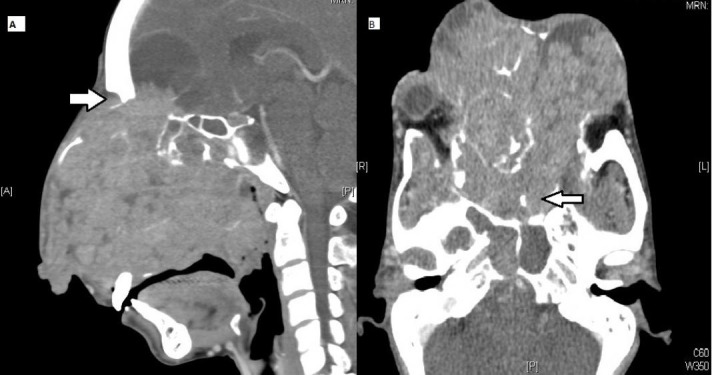
Pre-treatment CT (A) Sagittal section showing a large exophytic tumor with breach of the anterior cranial fossa (white arrow) and involvement of the sphenoid sinus. (B) Axial section demonstrating displacement of the orbit and invasion of the nasopharynx (white arrow). CT: Computed tomography.

A computed tomography (CT) scan of the face demonstrated an extensive bulky solid enhancing tumor with cystic areas involving the face, displacing bilateral orbits, and extending intracranially into the anterior cranial fossa (Figure 2).

Biopsy of the nasal lesion revealed multiple foci of squamous cell carcinoma on the background of Schneiderian papilloma. Staging positron-emission tomography-computed tomography (PET/CT) showed small volume lung metastases bilaterally.

**Figure 3:**
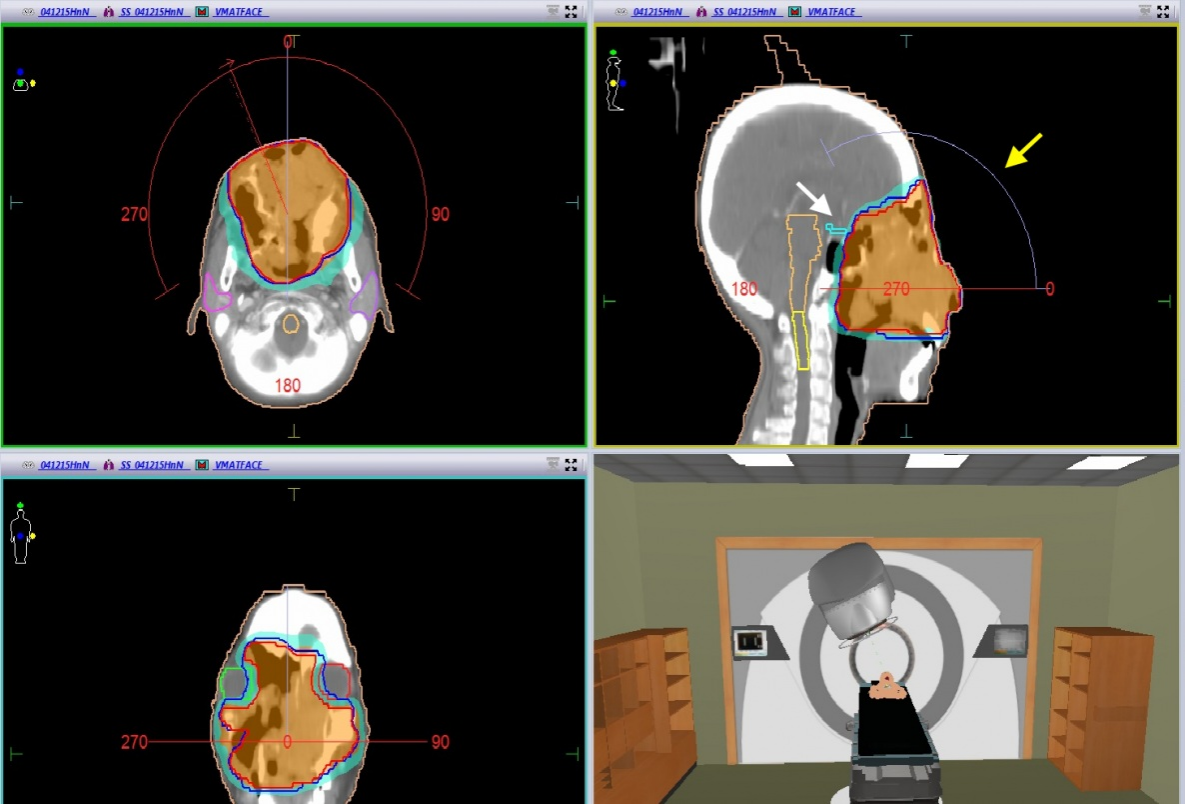
RT treatment plan at the time of progression VMAT plan using transverse and sagittal arcs (yellow arrow), while sparring the optic chiasm (white arrow) and both eyes. RT: Radiotherapy; VMAT: Volumetric modulated arc therapy.

She was commenced on multi-agent palliative chemotherapy with cisplatin, TS-1, and cetuximab. Three days after the first cycle of chemotherapy, the patient reported clinical improvement, but developed a torrential bleed from the primary tumor. Tumor embolization was deemed to have a high risk of necrosis and ischemic stroke. Urgent RT was instituted to achieve hemostasis. The CT-simulation could not be performed, as the patient was unable to tolerate the supine position (due to intractable coughing and facial pressure from the tumor). Eight Gy in two fractions over two consecutive days were delivered using opposed lateral fields, based on clinical mark-up, while the patient was sitting up. Successful hemostasis was achieved, and the patient was able to receive five further cycles of chemotherapy. She was referred back for further RT six months later, upon signs of local progression. Forty Gy in 15 fractions were delivered using a non-coplanar volumetric modulated arc therapy (VMAT) technique to reduce the cumulative dose to the retina and optic apparatus (Figure 3).

**Figure 4:**
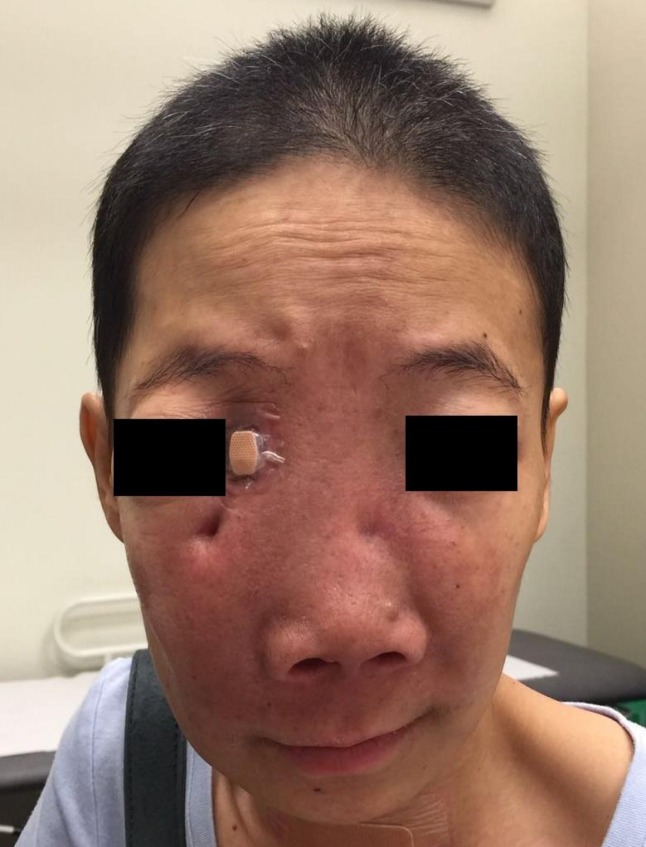
Post-treatment clinical photograph Displacement of the orbits was markedly reduced six weeks post RT. RT: Radiotherapy.

**Figure 5:**
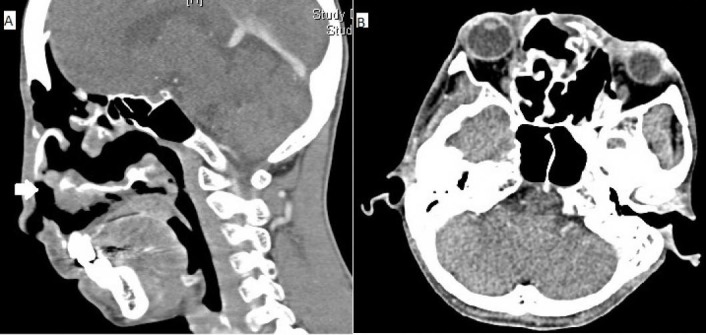
Post-treatment CT performed six weeks post RT (A) Sagittal section showing near complete resolution of the solid components of the tumor, with significant improvement in the nasal cavity (white arrow). (B) Axial section showing medialization of the orbits. Computed tomography (CT); Radiotherapy (RT).

 She achieved excellent clinical (Figure 4) and radiological responses (Figure 5) to RT. 

The patient had controlled disease for six months post treatment; however, her facial tumor and lung metastases began to progress thereafter. She was then treated with immunotherapy and presently has no further disease progression.

## Discussion

Malignant transformation of inverted papilloma is rare (10–11%). Unresectable cases are managed with RT and chemotherapy [5]. Tumor bleed can occur spontaneously but has also been associated with anti-angiogenic agents (e.g. bevacizumab) [6]. We hypothesize this patient’s bleed was precipitated by a rapid tumor shrinkage secondary to chemotherapy initiation. The circumstances of this case necessitated life-saving treatment without CT planning. RT has been described to have an excellent hemostatic efficacy [7]. Larger fraction sizes, above 2 Gy, are likely to play a role in hemostasis, possibly through activation of the ceramide pathway [8]. We demonstrate an excellent symptom palliation with multi-agent chemotherapy and advanced RT planning techniques. 

## Conclusions

A short course of RT (e.g. two fractions) may be enough for hemostasis, and large fraction sizes are likely to be beneficial. RT and chemotherapy offer a good response in unresectable metastatic malignant-inverted papillomas.
